# Female Patients with Pneumonia on Intensive Care Unit Are under Risk of Fatal Outcome

**DOI:** 10.3390/medicina58060827

**Published:** 2022-06-19

**Authors:** Mareike Kristina Koerber, Sarah Agaoglu, Anna Bichmann, Sascha Tafelski, Irit Nachtigall

**Affiliations:** 1 Department of Anesthesiology and Intensive Care Medicine, Campus Charité Mitte and Campus Virchow-Klinikum, Corporate Member of Freie Universität Berlin and Humboldt-Universität zu Berlin, Charité Universitaetsmedizin Berlin, Charitéplatz 1, 10117 Berlin, Germany; sarah.agaoglu@charite.de (S.A.); anna.bichmann@charite.de (A.B.); sascha.tafelski@charite.de (S.T.); irit.nachtigall@helios-gesundheit.de (I.N.); 2 Infectious Diseases and Infection Prevention, Helios Klinikum Emil-Von Behring and Region East, Walterhöferstr. 11, 14165 Berlin, Germany

**Keywords:** sex, pneumonia, ICU mortality

## Abstract

*Background and Objectives:* The impact of sex on mortality in patients with pneumonia requiring intensive care unit (ICU) treatment is still a controversial discussion, with studies providing heterogeneous results. The reasons for sex differences are widespread, including hormonal, immunologic and therapeutic approaches. This study’s aim was to evaluate sex-related differences in the mortality of ICU patients with pneumonia. *Material and Methods:* A prospective observational clinical trial was performed at Charité University Hospital in Berlin. Inclusion criteria were a diagnosis of pneumonia and a treatment period of over 24 h on ICU. A total of 436 mainly postoperative patients were included. *Results*: Out of 436 patients, 166 (38.1%) were female and 270 (61.9%) were male. Significant differences in their SOFA scores on admission, presence of immunosuppression and diagnosed cardiovascular disease were observed. Male patients were administered more types of antibiotics per day (*p* = 0.028) at significantly higher daily costs (in Euros) per applied anti-infective drug (*p* = 0.003). Mortalities on ICU were 34 (20.5%) in females and 39 (14.4%) in males (*p* = 0.113), before correcting for differences in patient characteristics using logistic regression analysis, and afterwards, the female sex showed an increased risk of ICU mortality with an OR of 1.775 (1.029–3.062, *p* = 0.039). *Conclusions*: ICU mortality was significantly higher in female patients with pneumonia. The identification of sex-specific differences is important to increase awareness among clinicians and allow resource allocation. The impact of sex on illness severity, sex differences in infectious diseases and the consequences on treatment need to be elucidated in the future.

## 1. Introduction

The COVID-19 pandemic has reignited a longstanding discussion about sex differences in infectious diseases. Every type of pneumonia necessitating intensive care unit (ICU) treatment is a life-threatening disease for patients. The outcome depends on multiple factors of differing importance, such as age and preexisting illnesses. Sex as an influencing factor has been discussed as another issue with inconsistent results, which might be due to various study cohorts and settings. Sharpe et al. report a lower incidence of ventilator associated pneumonia (VAP) in female patients after trauma but with a higher mortality [1]. This is partly consistent with Gannon et al., who identified male patients as being at higher risk for VAP, but found no differences in mortality [2]. Kollef et al. report a higher mortality in females with a need for mechanical ventilation [3]. An earlier publication from our group showed an increased mortality for females with sepsis [4]. Nachtigall et al. found a higher mortality risk in hospitalized male patients older than 60 years with COVID-19 [5]. In contrast, Ponce-Alonso et al. found no effect of sex on mortality in patients with sepsis [6]. The effect of sex on patient treatment in emergency departments and ICU is measurable and was demonstrated by Valentin et al. for therapeutic interventions and outcomes in the critically ill [7,8,9]. Reasons for different survival probabilities might be sex hormones, genetics, social gender implications such as smoking, and different intensity levels of care [10,11,12].

Sex hormones are involved in cardiovascular function, G protein-coupled estrogen receptors, toll-like receptors and leukocyte-platelet aggregate markers [13]. The immune response of females and males shows fundamental differences throughout a lifetime [14]. Males show a more intense inflammatory reaction which can influence disease severity. This results in different a function and response of the innate and adaptive immunity, leading to a diverging susceptibility to different germs and autoimmune diseases. Estrogen is known to induce a more efficient immune response to some pathogens and affects immune cell activity [14,15]. Clarity of the sex impact in those with pneumonia needing ICU treatment may improve vigilance for patients at higher risk, and thus cause lower mortality. This study investigated the influence of sex on mortality of ICU patients with pneumonia.

## 2. Materials and Methods

### 2.1. Study Design, Location and Patients

**Figure 1:**
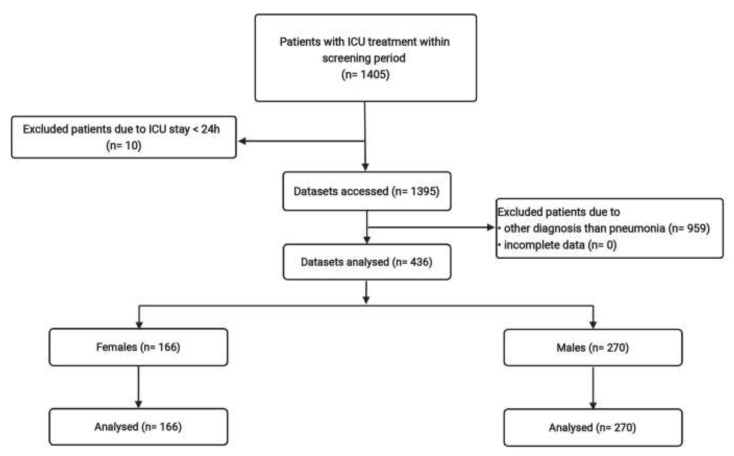
A total of 1395 surgical and non-surgical patients were included in the primary analysis of the first study.

All methods were performed in accordance with the relevant guidelines and regulations. This study was approved by the Charité Ethics board, no. (EA1/127/07). The Charité Ethics board has waived the need for informed consent for the current study. The following data are gathered from a secondary analysis of a prospective observational clinical study conducted between 2006 and 2010 at Charité University Hospital in Berlin, Germany [4]. For this purpose, initially, a total of 1395 surgical and non-surgical patients were included in the primary analysis of the first study (Figure 1). From this study population, a total of 436 patients were 18 years-of-age or older with a diagnosis of pneumonia and an admission duration lasting over 24 h to one of five anesthesiologically led ICUs, and were included in this secondary analysis. Hence, the patient population was a mixture of predominantly postoperative admissions ranging from cardiosurgical, neurosurgical, general, obstetric, head and neck interventions, polytrauma patients, to patients with neurological disorders or an adult respiratory distress syndrome (ARDS). The investigation was divided into four sections for the whole follow-up period of 320 days from admission. The diagnosis of pneumonia was via clinical examination and radiologic examination in correlation with laboratory and microbiologic results.

### 2.2. Variables

Patient baseline characteristics including sex, age, comorbidities, SAPS-II-, SOFA-, TISS-28 scores, clinical and laboratory variables, preexisting medication, immunosuppression, mechanical ventilation parameters were extracted from the patient data management system (PDMS) COPRA (Computer Organized Patient Report Assistant) Version 6–10 and MedVision version 3.38. Immunosuppression was defined as long-term corticosteroids administration above 7.5 mg prednisolone daily or other immunosuppressive medication, a known HIV infection, leukemia or previous chemotherapy. 

### 2.3. Statistical Analysis

Binary parameters and their distribution are shown as number (*n*) and percentage (%). Non-normally distributed or ordinal parameters are expressed as median and in an interquartile range (IQR; 25–75%). Normally distributed, metric parameters are shown as mean values (mv) with standard deviation (±SD). Normal distribution was evaluated graphically and by Shapiro–Wilk test. For statistical significance testing, a two-sided *p*-value of *p* = 0.05 was applied. Univariate analysis of binary parameters was performed with χ^2^-tests. Non-normal distributed metric or ordinal variables were tested with non-parametric Mann–Whitney-tests; in the case of normal distribution, significance level was calculated with a *t*-test. To assess the independent influence of sex on ICU mortality as the primary outcome parameter, we used logistic regression analyses to describe the relationship between parameters. Therefore, univariate regression analyses described parameters significantly associated with ICU mortality as a dependent variable. In a second step, a multivariate logistic regression analysis was performed with resulting parameters (threshold: *p* < 0.1) including sex as co-variable. Due to multicollinearity between ICU admission scores, we chose to limit analysis to SOFA score to include organ dysfunction into the model. The quality of the applied mathematical model was evaluated with the Homer–Lemeshow test. Data analysis was performed with PASW Version 18 (SPSS Inc. 1998–2010, Chicago, IL 60606, USA) and SAS (Version 9.1, SAS Institute Inc. 2003, Cary, NC, USA). 

## 3. Results

### 3.1. Basic Characteristics

A total of 436 patients were included for analysis, of which 166 were female and 270 were male. Basic parameters differed significantly for initial median SOFA score, immunosuppression and preexisting cardiac illness (Table 1). The main forms of pneumonia, community-acquired pneumonia (CAP), hospital-acquired pneumonia (HAP) and ventilator-associated pneumonia (VAP), were distributed equally in both groups. HAP was the most usual form, with 61% for females and males, followed by VAP and CAP (Table 1).

### 3.2. Differences of Patient-Centered Care

All main key indicators for comparison of patient-centered care, such as adherence to standard operating procedure (SOP), microbiologic and radiologic diagnostic, were not significantly different except for the antibiotic therapy (Table 2).

Male patients received a significantly higher number of different antibiotics per day and had a significantly higher incidence of calculated antibiotic therapy per day. The multivariate logistic regression analysis showed a significant association of age with ICU mortality. The same was seen with all admission scores (SAPS II, SOFA and TISS-28), preexisting pulmonary illness, nosocomial and ventilator-associated pneumonia (Table 3).

## 4. Discussion

The main finding of this prospective study is that mortality in pneumonia requiring ICU admission is influenced significantly by sex, with a higher mortality in females. Multivariate analysis revealed a higher mortality in females, which was undetected in the univariate analysis. This might be the reason that, so far, this finding is underreported, thus makes it difficult to compare to previously published results.

Sexes were not equally distributed with 38.1% females and 61.9% males. Nevertheless, this correlates with previously published ICU studies in which the female/male ratio is described as approximately 1:2 [1,16,17]. This was further investigated by Zettersen et al. who sent identical case descriptions to physicians labeled Jane or John [18]. They found no differences between female- or male-labeled cases for the decision for ICU admission, although this might be different in a real-life setting. The reason for this is most likely the higher incidence of preexisting medical conditions and not a sex-biased admission decision. This also appears to be contributing to the higher SOFA scores on ICU admission and the prevalence of preexisting cardiovascular disease in the male group. The SOFA score has been shown to be applicable to both sexes [19]. Female patients had higher rates of immunosuppression, as published in several rheumatologic studies [20,21].

Besides the hormonal impact on mortality, treatment of pneumonia is another major influencing factor on mortality. Our results found significantly more administration of mechanical ventilation in the univariate analysis in the male group. This could be explained by the higher proportion of pulmonary comorbidities and the higher initial SOFA score in men as mentioned above in the baseline characteristics. Epstein et al. did not find higher hospital mortality in females in the group of all ventilated medical ICU patients (not ventilated due to pneumonia only), although they performed a multivariate analysis adjusted for age, severity-of-illness scoring, comorbidities, and indications for mechanical ventilation [22]. This is consistent with the study of critically ill patients with nosocomial infections by Combes et al. and Caceres et al., who focused on patients with HAP [17,23]. In contrast, the need for mechanical ventilation was found to increase females’ mortality in an investigation by Kollef et al. within a mixed population of surgical and non-surgical patients [3].

Mechanical ventilation combined with more targeted antibiotic therapy suggests a more determined approach in pneumonia treatment. This is consistent with Pietropaoli et al., who found a higher risk for in-hospital mortality in females with sepsis and septic shock, and recognized differences in some aspects of ICU treatment as more mechanical ventilation, dialysis and prophylactic anticoagulation in men [24].

The difference in mortality was observed although we found no relevant differences in baseline characteristics, except age, immune suppression or cardiovascular disease. An obvious explanation, as mentioned in the introduction, could be the effect of estrogens. Estrogens are well known to improve the immune reaction to pathogens, e.g., via production of a higher amount of antibodies [25], but also increase the risk of autoimmune diseases [26,27,28,29]. The estrogen effect, though, has not been described for postmenopausal females, which represent the majority of our study population, having a median age of 68 years (57–77) [30]. The current COVID-19 pandemic has given deep insights on the sex impact on pneumonia survival [31]. Channappanavar et al. showed the negative effect of lowered estrogen levels in mice with SARS-CoV-2 [32]. Another study of mice detected higher proinflammatory cytokine levels in male mice during infection with *Streptococcus pneumoniae* [33]. The mortality advantage or disadvantage due to different immune response for males or females depends on the kind of pathogen causing different types of pneumonia: for example, VAP/HAP being most likely caused by bacteria [34,35].

Another approach to explain higher mortality could be the influence of sex on the interaction of medical staff and patients [36]. This has been shown for cardiovascular diseases [12,13]. Johnston et al. report less use of acute reperfusion therapy in females with ST-elevation myocardial infarction over the age of 60 years [11]. Samuelsson et al. reported in 2015 a higher nurse workload in men during ICU treatment, but no mortality disadvantage for females under the age of 45 years [37]. The significantly higher number of antibiotics in male patients is not necessarily an advantage [38]. A higher amount of care e.g., invasive procedures and increased care intensity, did not result in an improved outcome, as shown by Valentin et al. [7]. Despite this, the likeliness of ICU admission implies a shortage of medical care in females, which could also explain the higher mortality in this group [36].

Univariate analysis did not find a significant influence of sex on mortality. In the multivariate analysis, mortality was shown to be influenced by female sex, age and SOFA score on ICU admission. This suggests that if only univariate analysis is performed, the influence of factors such as fungus in our study are overestimated and do not allow a reliable interpretation of the data. The fact that females have a higher mortality in pneumonia with ICU admission, although their SOFA score is lower, leads to the question as to whether the SOFA score is applicable to both sexes.

## 5. Limitations

This study is a prospective observational study without randomization or blinding that did not measure social interaction effects between patients and medical staff. Although we assume most of the included females to be postmenopausal due to age, we did not measure sex hormone levels, which might have influenced the study results due to greater data inaccuracy [39]. The primary endpoint was ICU mortality and this did not include in-hospital mortality or mortality after discharge. In addition, a higher number of study patients might have allowed a more detailed sub-analysis of different pathogens or types of pneumonia and their impacts on patient outcome.

## 6. Conclusions

The effect of sex on ICU mortality during treatment for pneumonia is not yet completely understood. Our data show that it is essential to use customized statistical models to detect all relevant effects of sex; otherwise, these effects could remain undisclosed. In our study, there is evidence that the female sex is associated with higher mortality in pneumonia requiring intensive care treatment. Finally, our research data should increase scientists’ and clinicians’ vigilance and improve patient outcome irrespective of their sex.

## Figures and Tables

**Table 1:** Basic characteristics, distribution of preexisting illness and type of pneumonia.

Variable	Female	Male	*p*-Value
	*N* = 166	*N* = 270	
Age in years ^a^	65.1 ± 14.8	62.4 ± 15.3	0.116
Median ^b^	68 (57–77)	67 (52–74)	
Initial SAPS Score ^a^	42.8 ± 18.2	42.2 ± 15.8	0.899
Median ^b^	41 (29–54.3)	41 (31–53)	
Initial SOFA Score ^a^	5.5 ± 4	6.5 ± 4.1	**0.011**
Median ^b^	5.5 (2–8)	6 (4–9)	
Initial TISS-28 Score ^a^	35.6 ± 11.1	36.9 ± 11.1	0.317
Median ^b^	36 (27.8–44.2)	36.5 (28–44.3)	
Immune supression	21 (12.7)	18 (6.7)	**0.039**
Cardiovascular illness	63 (38)	143 (53)	**0.003**
Pulmonary illness	39 (23.5)	58 (21.5)	0.637
Chronic liver disease	17 (10.2)	31 (11.5)	0.754
Chronische kidney insufficiency	37 (22.3)	68 (25.2)	0.564
Diabetes mellitus	85 (51.2)	114 (42.2)	0.075
**Type of pneumonia *n* (%)**			
CAP (%)	27 (16.3)	33 (12.2)	0.254
HAP (%)	102 (61.4)	167 (61.9)	>0.999
VAP (%)	48 (28.9)	92 (34.1)	0.291
**Germ spectrum *n* (%)**			
Fungi	57 (34.3)	101 (37.4)	0.539
Germ with enhanced resistence potential (%)	31 (18.7)	51 (18.9)	>0.999
Gramnegative germ (%)	58 (34.9)	123 (45.6)	**0.035**

^a^ MV ± SD: Mean Value ± Standard Deviation. ^b^ (IQR; 25–75%): 25–75th Quartile. The bold marks significant results.

**Table 2:** Differences of patient-centered care with emphasis on adherence to SOP, antibiotic therapy, mechanical ventilation, microbiologic and radiologic diagnostic.

Variable	Female	Male	*p*-Value
	*n* = 166	*n* = 270	
Adherence to SOP ^a^	63.5 ± 34.6	67.2 ± 30.1	0.531
Median ^b^	70 (40–100)	71 (47–100)	
Daily dose of antibiotics in substances per day ^a^	1.1 ± 0.7	1.3 ± 0.7	**0.028**
Median ^b^	1 (0.6–1.5)	1.2 (0.8–1.8)	
Antibiotic-free days ^a^	33.7 ± 28.7	28.5 ± 25.0	0.104
Median ^b^	30.6 (9.1–50.0)	25.0 (7.3–44.5)	
Operated microbiologic diagnostic (sum) ^a^	6 ± 7.3	6.9 ± 8.3	0.197
Median ^b^	4 (1–9)	4 (1–11)	
Operated radiologic diagnostic (sum) ^a^	7.4 ± 6.7	8.4 ± 7.1	0.077
Median ^b^	5 (3–9)	6 (3–11)	
Calculated antibiotic/LOS ^g^ in d ^a, h^	0.5 ± 0.3	0.5 ± 0.3	0.146
Medianb	0.5 (0.3–0.8)	0.4 (0.2–0.7)	
Targeted antibiotic therapy LOS ^g^ in d ^a, h^	0.2 ± 0.3	0.3 ± 0.3	**0.016**
Median ^b^	0 (0–0.3)	0.1(0–0.5)	
ICU mortality (%)	34 (20.5)	39 (14.4)	0.113
ICU LOS ^g^ in d ^a^	17.9 ± 14.4	18.4 ± 15.6	0.891
Median ^b^	13 (8–25)	14 (7–24)	
Mechanical ventilation (%)	142 (85.5)	252 (93.3)	**0.011**
Durance of mechanical ventilation in h ^a^	295 ± 349	306 ± 489	0.919
Median ^b^	161 (30–466)	157 (36–392)	
ICU mortality (%)	34 (20.5)	39 (14.4)	0.113

^a^ MV ± SD: Mean Value ± Standard Deviation. ^b^ (IQR; 25–75%): 25–75th Quartile. ^g^ LOS: length of stay. ^h^ d: die, days. The bold marks significant results.

**Table 3:** Logistic regression analysis with intensive care mortality as dependent variable.

	Univariate Analysis ^c^		Multivariate Analysis ^d^	
Variable	OR (95% CI)	*p*-Value	OR (95% CI)	*p*-Value
Sex (♀ Female vs. ♂ Male)	0.655 (0.395–1.088)	0.103	1.775 (1.029–3.062)	0.039
Age	1.018 (0.0996–1037)	0.055	1.025 (1.006–1.046)	0.014
Scores at Hospital Admittance				
Initial SAPS	1.033 (1.017–1.049)	<0.001		
Initial SOFA	1.128 (1.061–1.200)	<0.001	1.139 (1.063–1.220)	<0.001
Initial TISS	1.060 (1.034–1.086)	<0.001		
Comorbidities				
Immune suppression	1.097 (0.464–2.591)	0.833		
Cardiovascular comorbidities	0.968 (0.585–1.602)	0.900		
Pulmonary comorbidities	1.946 (1.121–3.378)	0.018	1.598 (0.886–2.883)	0.119
Type of Pneumonia				
Early and late onset HAP	0.580 (0.350–0.963)	0.0354	0.799 (0.390–1.633)	0.538
SCAP	0.860 (0.403–1.836)	0.697		
VAP	1.974 (1.182–3.297)	0.009	1.431 (0.672–3.048)	0.353
Coinfections				
Abdominal infections	1.750 (0.786–3.896)	0.171		
Urogenital infections	0.994 (0.423–2.334)	0.989		
Bone-/joint infections	1.513 (0.406–5.638)	0.537		
Endocarditis	0.994 (0.213–4.636)	0.994		
Wound/ soft tissue infections (%)	1.864 (0.956–3.632)	0.067	1.816 (0.850–3.878)	0.123
Meningitis	0.702 (0.156–3.158)	0.645		
Focus not identified	0.896 (0.361–2.222)	0.812		
Catheter infection	1.644 (0.675–4.005)	0.274		
Pseudomembranousus Colitis	1.250 (0.260–6.010)	0.781		
Germ Spectrum				
Fungi	1.680 (1.011–2.794)	0.045	1.130 (0.643–1.986)	0.671
Germ with enhanced resistance potential (%)	1.675 (0.930–3.020)	0.086	1.145 (0.581–2.258)	0.696
Gramnegative germ (%)	1.369 (0.826–2.268)	0.223		
SOP-Adherence				
SOP adherence > 70 (%)	0.768 (0.464–1.272)	0.305		

^c^ Left column: Univariate logistic regression for explanation of the influence of different parameters on ICU mortality. ^d^ Right column: Multivariate logistic regression analysis with inclusion of parameters with significant association on intensive care mortality. Odds ratio (OR) and confidence interval (CI) are shown. Hosmer–Lemeshow test *p* = 0.202.

## Data Availability

The datasets used and analyzed during the current study are available from the corresponding author on reasonable request. The data were presented on the 7th of October 2020 at the 35th congress of the Deutsche Aerz-tinnenbund e.V. in Berlin, Germany.

## Abbreviations

CAP	Community-acquired pneumonia
COPRA	Computer Organized Patient Report Assistant
HAP	Hospital-acquired pneumonia
IQR	Interquartile range
ICU	Intensive care unit
PDMS	Patient data management system
SOFA	Sequential organ failure assessment
SD	Standard deviation
SOP	Standard operating procedure
VAP	Ventilator-associated pneumonia
